# Flowered Grain Quality and Phytochemical Content of Non-Conventional Maize Hybrids from the Mexican Subtropics across Three Growing Cycles

**DOI:** 10.3390/plants12142691

**Published:** 2023-07-19

**Authors:** Leticia García-Cruz, María Gricelda Vázquez-Carrillo, Ricardo Ernesto Preciado-Ortiz

**Affiliations:** 1 Laboratorio de Maíz, Campo Experimental Valle de México, Instituto Nacional de Investigaciones Forestales, Agrícolas y Pecuarias (INIFAP), Km 13.5 Carretera Los Reyes-Texcoco, Coatlinchán 56250, Estado de México, Mexico; letygc_1189@hotmail.com; 2 Programa de Mejoramiento Genético de Maíz, Campo Experimental del Bajío, Instituto Nacional de Investigaciones Forestales, Agrícolas y Pecuarias (INIFAP), Carretera Celaya-San Miguel Allende Km. 6.5, Celaya 38110, Guanajuato, Mexico; repreciado@yahoo.com

**Keywords:** Elotes Occidentales, total anthocyanin, total soluble phenols, volume of flowered grain

## Abstract

Development of non-conventional hybrids responds to the demand for the Elotes Occidentales land-race for production of pozole. The effect of growing cycle (2019, 2020, and 2021) on physical characteristics, flowered grain quality, and phytochemical content of two non-conventional hybrids of pozolero maize, as well as the effect of the presence or absence of pedicel, type of pollination (open and controlled, 2019), and parents (female and male, 2020) on flowered grain quality and content of phytochemical compounds, were evaluated. Size, hardness, color, total phenols, and anthocyanins in unprocessed grain were determined. Yield, volume, and puncture force were measured in flowered grain. Results were analyzed with a factorial arrangement in a completely randomized design. There were significant differences (*p* ≤ 0.05) in most of the variables studied by effect of crop cycle and hybrid. Non-conventional hybrids had large grains (40 g 100 grains^−1^), soft endosperm (flotation index > 60), pink-purple color, and phenol and anthocyanin contents similar to those reported for the Elotes Occidentales land-race. The presence or absence of the pedicel did not affect flowered grain quality. Controlled pollination favored anthocyanin synthesis. The female parent determined the anthocyanin content of non-conventional hybrids. Thermal processing reduced anthocyanins by 60%; however, they leached into the flowering broth, so that the pozole made from non-conventional hybrids can have improved nutraceutical value, relative to that of pozole made with Cacahuacintle land-race.

## 1. Introduction

Of the 64 maize races identified in Mexico [1], the races specialized for pozole preparation stand out. Pozole is dish that is only surpassed by ‘mole’ in volume of consumption [2]. Like many other foods of pre-Hispanic origin, Pozole is made with nixtamalized maize grains before obtaining the flowered grain. Nixtamalization consists of cooking the maize grain in water and calcium hydroxide, at varied times depending on the hardness of the grain [3]. During this stage, the grains undergo several changes that improve their nutritional value, since the content of calcium and resistant starch increases and there is a greater availability of iron and niacin. On the other hand, the content of phytic acid (a compound considered anti-nutritional) and mycotoxins is reduced [4]. Nixtamalization is a very strong thermal-alkaline treatment that leads to the degradation of a high percentage of anthocyanins in pigmented corn [5]; however, it has been reported that even after obtaining the flowered grain, anthocyanins are still present in the aleurone layer [6].

The base of pozole is flowered grain, which is preferably prepared with Cacahuacintle, Ancho, and Elotes Occidentales races. These races are produced in specific ecological niches, limiting their production. They have low yields (3.6 t ha^−1^) associated with problems of lodging, asynchrony between female and male flowering, grain rot, etc. [7]. Native maize races are of great importance because they are the key to overcoming the current difficulties in agriculture, since they are the basis for genetic improvement. The aim is to improve food security, face climate change, and make agriculture a profitable and sustainable system [8].

In Mexico, maize genetic improvement has focused mainly on the development of dents hybrids of white grain, which are mainly used for human consumption, in the preparation of more than 600 traditional dishes [9,10]. Additionally, high-protein quality maize [11]; popcorn [12] with high oil content; and pigmented, sweet, forage maize; etc. [13,14] are studied. In recent years, interest in pozolero maize has grown, and work has been conducted on its genetic improvement. Non-conventional hybrids (NCHs) have been developed from the Elotes Occidentales race, with better agronomic characteristics than the native [15].

The non-conventional hybrids (NCHs), which can be defined as a hybrid with at least one parent, are not an inbred line, such as open pollination varieties from the Elotes Occidentales ‘EO’ land-race. The heterosis presented in these NCHs favors an increase in of grain yield up to 12.4 t ha^−1^ and reduces the limitations of the original native, while maintaining the earliness and grain quality characteristics [15]. The ‘EO’ race is characterized by ears 15 to 20 cm long, cylindrical in shape and with a low number of rows [6,16]. The grains are large and mealy, with pink, pink-red, blue, and purple colorations [15].

The purple, red, and blue tones in grains of the ‘EO’ race present in new NCHs are due to anthocyanins in the aleurone layer [6]. Anthocyanins are pigments that have gained great relevance due to their antimicrobial, antimutagenic, anticancer, and anti-inflammatory properties [17].

Because of the nature of the color of ‘EO’ grains, the anthocyanins that have been identified are mainly derived from pelargonidin-3-O-glucoside [18]; however, the coloration originates from a mixture of compounds, and other anthocyanins that have been identified in smaller proportions in this race are derived from cyanidin-3-glucoside and peonidin-3-glucoside [16].

Pigmented maize has a price premium of 30–40% over white maize [10], and there is currently a trend towards preference for consumption of pigmented maize over white maize; thus, NCHs can be a better option in terms of nutraceutical quality than unpigmented maize. In this sense, the objectives of this study were to evaluate the effect of (1) growing cycle (GC) on physical characteristics, flowered grain quality, and phytochemical content of two NCHs of pozolero maize; (2) presence or absence of pedicel on flowered grain quality and phytochemical content; and (3) type of pollination (open and controlled, 2019) and parental components (female and male, 2020) on phytochemical content.

## 2. Results and Discussion

### 2.1. Physical Characterization of Grain

The analysis of variance (ANOVA) showed significant statistical difference (*p* ≤ 0.05) in most of the variables evaluated due to the effect of GC and NCHs (Table 1). The test weight of Pz2 was very stable during evaluation cycles; its value ranged from 73.1 to 73.9 kg hL^−1^. The hundred grains weight (HGW) was 36.4–44.5 g, values that qualify them as large grains (>38.0 g) [19]. In both variables, the highest values were found in hybrid Pz2. In the ‘EO’ race, HGW values between 27 and 64 g have been reported [6,20], which is consistent with what was found in this research. The flotation index (FI) varied from 58 to 93 floating grains. In the 2019 cycle, the grains had intermediate texture, while in 2020 and 2021 their endosperm had a very soft texture. The FI correlated significantly with the percentage of floury endosperm (EH) (r = 0.72; *p* > 0.01), corroborating the soft texture of these grains. The proportions of vitreous endosperm decreased (Table 1).

In the pedicel (PE) and pericarp (PER) fractions, crop year and hybrid did not cause significant differences, possibly indicating that these variables are less influenced by the environment, except for year 2020 when Pz1 had a lower % of pedicel than Pz2 (Table 1). PE and PER values for Pz1 and Pz2 hybrid agree with Preciado-Ortiz et al. [15] for NCHs. The reduced amount of PE (0.85–1.32%) in NCHs, relative to that in Cacahuacintle (2.4%) [21], could be due to genetic and environmental aspects as well as smaller grain size of NCHs.

The % of germ had few changes over the evaluation years with values from 10.4 to 11.4% (Table 1), results that were similar to those reported by Vázquez-Carrillo et al. [22] for a collection of the ‘EO’ race but lower than those reported by Preciado-Ortiz et al. [15] in NCHs.

Grain color was pink-purple with luminosity values between 41.87 and 50.43%, hue from 51.4 to 69.1°, and chroma from 11.7 to 14.2; the color was characteristic of ‘EO’ race; and the results were similar to those reported by Ballesteros Martínez et al. [6] for the same race.

### 2.2. Flowered Grain Quality

The ANOVA of the technological variables showed statistical difference (*p* ≤ 0.05) in the flowered factor (with and without pedicel). Significant differences were present in flowered grain moisture (FGM) and color variables (L*, hue, and chroma), while in the hybrid factor there were differences in all variables, except in dry matter loss (DML) (Table 2).

### 2.3. Effect of Type of Flowered

**Figure 1:**
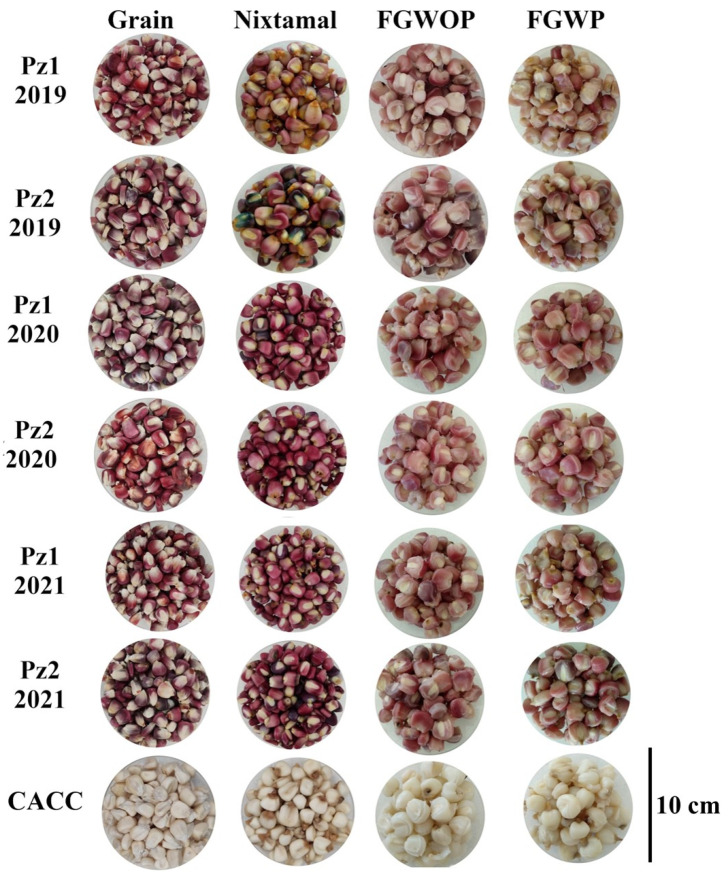
Appearance of raw grain, nixtamal, flowered grain without pedicel (FGWOP), and flowered grain with pedicel (FGWP) of two maize hybrids of the Elotes Occidentales race evaluated in three crop cycles, and a hybrid of the Cacahuacintle race.

Flowered grains without pedicel (FGWOP) had higher moisture content (62.2 ± 3.4%) than flowered grains with pedicel (FGWP) (61.1 ± 2.7%), suggesting that elimination of the pedicel may have facilitated the entry of water into the endosperm. However, it may also have been due to a concentration effect since with the pedicel present, there is a greater amount of dry matter, resulting in a lower moisture content. It was found that after flowering (almost three hours) the aleurone layer remained attached to the endosperm, which favors the retention of the grain components and preserves the pinkish color of this structure (Figure 1). Santiago-Ramos et al. [23] mention that the aleurone layer persists attached to the endosperm even after the alkaline and thermal conditions of nixtamalization and prevents losses of protein and starch. The color of FGWOP was lighter (48.0 ± 6.4%) but with less hue and purity (52.1 ± 15.8° and 14.28 ± 1.5), while lightness in the color of FGWP was reduced, and hue and purity of color increased (45.1 ± 6.4%; 56.6 ± 13.4° and 16.7 ± 1.6, respectively), a difference that is attributed to the dark color of the pedicel. The rest of the flowered grain (FG) technological variables were not affected by the presence or absence of pedicel (Table 2), results that could explain the use of de-headed ‘EO’ maize in the region where it is consumed. On the other hand, Preciado-Ortiz (2023; pers. comm.) reported that the presence of the PE in ‘EO’ flowered grain does not affect the palatable characteristics of flowered grain.

### 2.4. Effect of Genetic Materials

The NCHs grown in 2019 required the least time to flower (FT), and the rest of the genetic materials, including the Cacahuacintle control (CACC), required 160 and up to 205 min to flower (Table 3). Ballesteros Martínez et al. [6] reported similar values. The loss of dry matter (LDM), which includes solids in nejayote and those in washing and flowering water, was 6.88 to 7.71%, not statistically different from CACC. The high alkalinity of nejayote (pH > 12) promotes pericarp solubility; thus, it was in nixtamalization where the highest LDM was recorded, as Méndez-Lagunas et al. [24] also reported. Flowered grain moisture (FGM) ranged from 57.6 to 64.1%. The NCHs in the 2020 and 2021 cycles and CACC had the highest moisture content and were statistically different from hybrids Pz2-19 and Pz1-19C (Table 3). Flowered grain yield (FGY) ranged from 2.24 to 2.55 kg of flowered grain per kg of maize. Pz1-19 and Pz1-19C, with open and controlled pollination, were the lowest yielding and statistically different from the rest of the ‘EO’ hybrids and CACC. This difference was associated with a greater hardness of their grains (FI < 68). The largest volume of flowered grain (VFG) was found in the female parent ♀Pz1-20 (385.0 cm^3^ 100 g^−1^ maize) and was statistically equal to hybrids Pz1-20, Pz2-21, female ♀Pz2-20, and CACC. Hybrids grown in 2019 had the lowest volumes; they were also among those that required less FT and more force to be perforated, which is attributed to the greater hardness of their grains (FI < 70).

In the NCHs of 2019, which were the hardest grains, the flowered grain quality was diminished, due to a decrease in FGM, FGY, and VFG; they also required the greatest puncture force (PFFG). Controlled pollination (C) did not affect the technological characteristics of Pz1 maize, while Pz2-19C, with an average FI of 99, required less FT (150 min) and less PFFG (1.59 N). In addition, it reached a good FGM (60.3%), while the rest of the variables were similar to those of its homologous Pz2-19 with open pollination, with values between 1.14 and 1.61 N (Table 3). With the female parent of Pz1-20, the numerically highest VGF was produced (385 cm^3^ 100 g^−1^ maize). Similar values were reported for the Cacahuacintle race (388 cm^3^ 100 g^−1^ maize) [25], which required the least PFFG (1.14 N). These characteristics are associated with starch and the formation of the amylose–lipid complex, which gives greater stability to hydrated starch granules, imparting a spongy structure, associated with a soft texture and easy chewiness [26].

In the color parameters L*, hue, and chroma, there were significant statistical differences between NCHs and CACC, which was expected, since CACC maize is white (does not produce anthocyanins). On the other hand, NCHs presented pigmentation in the aleurone layer, which was maintained in the flowered grain (Figure 1), a result that supports the added value of NCHs, compared to that of white grains. The processing of NCHs (nixtamalization and flowering) reduced the color of the original grains, especially those harvested in 2019 (Figure 1).

### 2.5. Phytochemical Components

#### 2.5.1. Total Soluble Phenols (TSPs) and Total Anthocyanins (TAs) in Whole Grain

**Figure 2:**
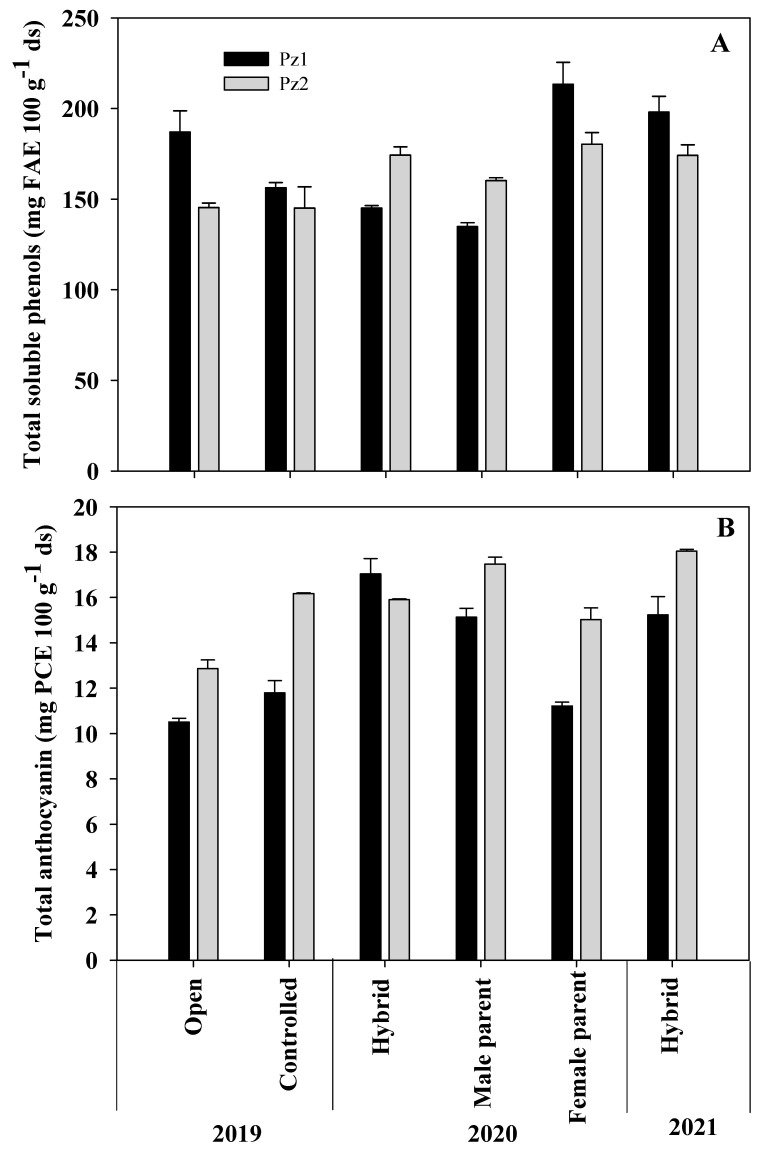
Total soluble phenol (**A**) and total anthocyanin (**B**) content of two hybrids of the Elotes Occidentales land-race and their parents, developed for the Mexican subtropical regions.

In the 2019 GC, Pz1 had the highest TSP content (187.1 mg FAE 100 g^−1^ ds), higher than Pz2 (145.3 mg FAE 100 g^−1^ ds). In TA, the behavior was reversed, and Pz2 had the highest content (12.9 mg PCE 100 g^−1^ ds) (Figure 2B). With controlled pollination, TSP was similar in both NCHs (Figure 2A). When comparing pollination types (cycle 2019), the TSP content in NCHs Pz1-19C (controlled pollination) decreased by 16.43%, while in Pz2 it remained at 145 mg FAE 100 g^−1^ ds. Total anthocyanin in Pz1-19C and Pz2-19C, produced with controlled pollination, increased by 12.2 and 25.4%, respectively. The effect of pollination type on anthocyanin content has not been evaluated; however, Kahriman et al. [27] found that carotenoid content increases significantly when produced with controlled pollination relative to that with open pollination. 

In the 2020 GC, the highest TSP contents were found in the male parents of both hybrids (215.3 and 180.32 mg FAE 100 g^−1^ ds), while the lowest values were found in the female parents (134.8 and 160.2 mg FAE 100 g^−1^ ds), and intermediate contents (145.0 and 174.5 mg FAE 100 g^−1^ ds) in the NCHs (Figure 2A). Both parents could have influenced the results obtained in the hybrids since Darrah et al. [28] mention that the pericarp has a maternal origin, while the germ has both female and male influence. Studies have determined that the highest concentration of TSP is found in the pericarp and germ [29]. In contrast, Ruiz-Torres et al. [30] found that the genetic effects of the female lines mainly determine the expression of phenolic compounds in white maize crosses. Regarding TA content, the highest concentration was found in the female line of ♀Pz2 (17.48 mg PCE 100 g^−1^ ds). In the Pz2 hybrid, it decreased to 15.9 mg PCE 100 g^−1^ ds, behavior that was reversed in the Pz1 hybrid, in which heterobeltiosis was observed with respect to this variable since it registered the highest TA content (17.04 mg PCE 100 g^−1^ ds). In both hybrids, their respective male parents had the lowest values (Figure 2B). The results found could be due to the fact that pigments were present only in the aleurone layer, and this structure has triploid cells (3*n*) in which one paternal and two maternal genes participate [31]; so, as previously reported, anthocyanin content in hybrids is determined by the parent that participates as female in the cross [15,30].

Regarding the behavior of hybrids in the evaluated cycles, in TSP content, the hybrid Pz2 was more stable, with contents of 145.3, 174.3, and 174.2 mg FAE 100 g^−1^ ds for the 2019, 2020, and 2021 cycles, respectively. In hybrid Pz1, TSP contents were 187.01, 145.0, and 198.0 mg FAE 100 g^−1^ ds for the same years (Figure 2A). Little has been reported on TSP content in ‘EO’ land-race samples. In this regard, Ballesteros Martínez et al. [6] reported values lower than those found in this work, ranging from 100 to 130 mg FAE g^−1^ ds, while Rocandio-Rodríguez et al. [32] reported 344 mg GAE 100 g^−1^ ds in a cross of Elotes Occidentales × Tuxpeño. Regarding TA content, a trend of interaction with the environment was observed across years, with values of 10.5, 17.0, and 15.2 mg PCE 100 g^−1^ ds for Pz1 in cycles 2019, 2020, and 2021, respectively, and 12.9, 15.9, and 18.1 mg PCE 100 g^−1^ ds for Pz2 in the same years (Figure 2B). In the 2019 and 2021 cycles, the Pz2 hybrid was superior to Pz1. The values found were similar to those reported by Broa Rojas et al. [33] in some ‘EO’ crosses, and Preciado-Ortiz et al. [15] in NCHs of the ‘EO’ race. The behavior of both hybrids in TSP and TA content in the evaluation cycles indicated good adaptability to the production area for which they were developed; in both cases they have shown upward behavior, which could indicate that better climatic conditions favored the synthesis of these phytochemicals. This contrasts with Mansilla et al. [34], who observed a decrease in phenol and anthocyanin contents in purple maize over four growing cycles, possibly due to poor adaptation of the seed to the growing area, as they used a mixture of introduced seeds from several countries.

#### 2.5.2. Total Soluble Phenols and Total Anthocyanin Content in Flowered Grain, Nejayote, Wash Water, and Flowering Broth

**Figure 3:**
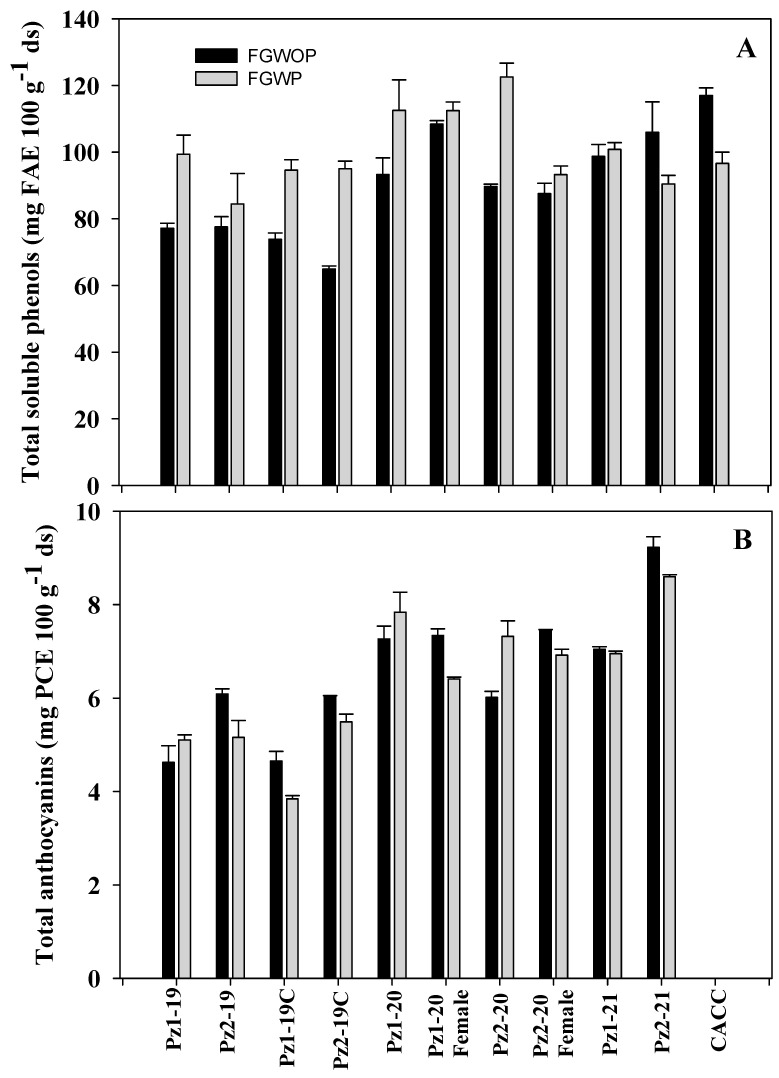
Contents of total soluble phenols (**A**) and total anthocyanins (**B**) in flowered grain without pedicel (FGWOP) and with pedicel (FGWP) of NCHs of ‘EO’ maize with open and controlled pollination (2019) and its female parents (2020), evaluated in 2019, 2020, and 2021 growing cycles in Celaya, Guanajuato, Mexico.

A statistically significant difference (*p* ≤ 0.05) was found between flowered grain with and without pedicel in all evaluated variables. The highest concentration of TSP was for flowered grains with pedicel (Figure 3A). The TSP content in FG ranged from 64.96 to 116.99 mg FAE 100 g^−1^ dm.

This behavior can be explained since phenolic acids derived from hydroxycinnamic acid are bound to lignocellulosic material [35]. Little information is available on phenolic content in flowered grain. In this regard, Peralta-Veran et al. [36] found contents of 50–71 mg GAE 100 g^−1^ ds, which are lower than those found in the present study. However, Ballesteros Martínez et al. [6] found higher contents in flowered grain of maize populations of the Elotes Occidentales race. The phenolic content of unprocessed grain decreased in flowered grain from 16.2 to 49%, with the hybrids that had the pedicel in the flowered grain suffering the losses due to temperature and prolonged flowering times. On average, in flowered grains without pedicel (FGWOP), there was a decrease of 48%, while in FGWP the reduction was 36.9%. In both types of flowered grain, TSP losses were lower than those reported by Peralta-Veran et al. [36] who reported losses of 70%. It is interesting to mention that the genetic materials with the lowest TSP content in grain (Pz1-20 and ♀Pz1-20) were those that retained the most of these compounds in flowered grain either with or without pedicel; Peralta-Veran et al. [36] observed similar behavior.

Several studies mention that the highest content of phenolic compounds is found in the pericarp followed by the germ, while a minimal amount is found in the endosperm [29,37]; however, these compounds have also been reported in the cell wall of aleurone cells [38]. In the flowered grain, the pericarp was no longer present, so it is probably the TSP of the aleurone layer and the germ that is being quantified, as well as that in the pedicel when present.

The total anthocyanin (TA) content in flowered grain ranged from 3.84 to 9.23 mg PCE 100 g^−1^ ds, with no statistical difference (*p* ≤ 0.05) between the grains with or without pedicel (Figure 3). The differences between one type of grain or another may be due to a dilution effect, since the presence of the pedicel translates into a higher proportion of dry matter. The same pattern in raw grain was also observed. The 2019 hybrids had lower concentrations than NCHs grown during 2020 and 2021, possibly due to harder grains (IF of 58 and 68%) since it has been reported that anthocyanin content decreases in hybrid maize due to an increase in hardness compared to native maize, as TA content is related to endosperm softness [39]. During processing from raw grain to flowered grain, TA losses ranged from 47.8 to 68%. Once again, it was evident that hybrids with higher TA content had greater losses than those with lower TA content. TA contents were slightly higher than those found by Ballesteros Martínez et al. [6], who reported values of 4 to 6.37 mg EPC 100 g^−1^ ds in flowered grain, with average losses of 71%.

The correlation between FI and TA showed that softer grains had higher anthocyanin content (Table 4). This suggests that after processing (flowering), the aleurone layer adhered more strongly to the vitreous endosperm, which could favor its retention, in contrast to what was observed in grains with floury endosperm, where these phytochemicals were lost in smaller amounts (Figure 2 and Figure 3). However, more studies are needed in this regard. Total anthocyanins in FGWP and FGWOP showed a high negative correlation with hue and L of flowered grain with and without pedicel (Table 5), which demonstrates that they are responsible for grain coloration. The higher the TA values, the less lightness the grains will have, and the hue values will be closer to zero, where the red color is found in the L*, C, h color space. In unprocessed grain, no negative correlation of TA with L and hue was observed, but this is possibly because the reading was on the pericarp layer, which interferes with the expression of the color of the aleurone layer.

Complete elimination of the pericarp and subsequent flowering significantly reduced phenolic compounds in flowered grain (Figure 2A). The alkaline conditions and nixtamalization temperature favor the leaching of phenolic compounds into the nejayote (TSP_n_), where the highest concentration of TSP_n_ was found, with values from 297.2 to 324.9 mg FAE L^−1^, while in the washing water (TSP_w_) values were from 179.8 to 230.0 mg FAE L^−1^ (Table 5). These results were lower than those reported by other authors [24,40], possibly due to the type of maize and low TSP content in raw grain. In broth of FG without pedicel, (TSP_b_) phenol contents were between 156.6 and209.3 mg FAE L^−1^. It was observed that in broth of FG with pedicel there was a higher concentration of these metabolites, 197.6–263.9 mg FAE L^−1^, which could be due to the fact that phenols present in the lignocellulosic material of the pedicel were leached into the flowering broth, since the samples with the highest TSP content were those that had the highest proportion of pedicel. Flowering time was not modified by the presence or absence of the pedicel (Figure 2, Table 1).

The anthocyanin content in flowering broth ranged from 4.25 to 12.76 mg PCE L^−1^. The effect of flowering time was evident, since hybrids that required more FT (2020 and 2021) presented the greatest leaching of anthocyanins from grain to broth (Table 5). It was also observed that FGWOP leached more anthocyanins into the flowering broth than their counterparts with pedicel. However, this could also be due to flowering time since FG without pedicel required more time than FG with pedicel (Table 5). In flowered grains, endosperm flowering explosion occurs at the apical or lateral part. Phytochemical compounds that are leached into flowered broth are not considered losses since in the preparation of the dish called pozole, the flowering broth is consumed as well as flowered grains. Therefore, making pozole with pigmented grains could be an option for consuming foods with nutraceutical value since it has been reported that maize anthocyanins have anticancer activity and cardioprotective, antimicrobial, antimutagenic, and antidiabetic effects, to mention a few [17,41].

## 3. Materials and Methods

### 3.1. Non-Conventional Maize Hybrids

The non-conventional maize hybrids (NCHs) Pz1 and Pz2, which include the ‘EO’ land-race in their genealogy, were studied. In 2019, 2020, and 2021 spring–summer growing season, at the Campo Experimental Bajío, Instituto Nacional de Investigaciones Forestales, Agrícolas y Pecuarias (INIFAP), NCHs with open pollination were evaluated. In 2019, NCHs with controlled pollination were also evaluated (Pz1-19C y PZ2-19C), and in the 2020 growing cycle the male and female parents of the two hybrids were included. To distinguish the hybrids in the different growing cycles, a hyphen and the year were added (Pz1-19, Pz1-20, Pz1-21, likewise for the hybrid Pz2) As a control to evaluate the quality of flowered grain, a commercial Cacahuacintle land-race (CACC) white grain maize was used, as it is most commonly used for this purpose in central Mexico [2]

### 3.2. Physical Characteristics of Grain

The variables test weight (TW), the hundred grains weight (HGW), and grain hardness measured using the flotation index (FI) and the percentages of pedicel, pericarp, germ, and floury and vitreous endosperm were determined via manual dissection according to the standard NMX-034/1 [19]. Color in unprocessed and flowered grain was measured with a Hunter Lab MiniScanXEPlus^®^ colorimeter (Reston, VA, USA) from which L*, a*, and b* parameters were obtained, and hue angle ($\mathrm{hue}=\arctan(\frac{b}{a})$) and saturation index ($\mathrm{chroma}={\left(a^{2}+b^{2}\right)}^{1/2}$) were determined [42].

### 3.3. Nixtamalization and Obtaining Flowered Grain

A 100 g sample of maize free of pests and damage was nixtamalized with 250 mL of water and 1% Ca(OH)_2_ with respect to the amount of grain. The nixtamalization time was assigned according to FI, following NMX-034/1 [19]. After cooking, it was left to rest for 16 h. After this time, it was strained to remove the nejayote, and the nixtamal was washed to remove the pericarp. The pedicel was manually removed (de-headed). The washed and de-headed nixtamal was subjected to a second cooking to obtain flowered grain, for which 250 mL of water was added. Flowering time was measured from when the samples started to boil until 60% of a random sample of 10 grains had flowered. The grains were separated from the cooking liquor, and the volume of flowered grain (VGF) was measured; the result is reported in cm^3^ 100 g^−1^ maize. A 50 mL sample was taken of nejayote (maize cooking liquor), washing water, and flowered broth and evaporated in an oven at 130 °C. The percentage of dry matter loss (DML) was obtained by adding the solids from the three liquids. The technological variables of flowered grain were studied in grain with and without pedicel.

### 3.4. Quality of Flowered Grain

In flowered grain (FG), the following was quantified: yield (YGF) expressed in kg FG per kg maize; moisture (FGM) following method 44-15.02 of the American Association of Cereal Chemistry [43]; and puncture force (PF) obtained on a Brook-field^®^ texturometer model CT3 (Middleboro, MA, USA), using a needle-shaped strut. Results are reported in Newtons.

### 3.5. Phytochemical Components

The contents of total soluble phenols (TSP) and total anthocyanins (TA) in raw maize, flowered grain, nejayote, washing water, and flowered broth were analyzed.

#### 3.5.1. Obtaining the Extract

In the case of solid samples, 100 mg of flour previously defatted with petroleum ether for 8 h in Goldfisch Labconco^®^ (Kansas, MO, USA) equipment was weighed. In the case of nejayote, washing water, and flowering broth, 1 mL of a homogenized sample was taken and placed in a 2 mL capacity Eppendorf^®^ tube. In both cases, it was gauged to 2 mL with 1% TFA prepared in 80% methanol. The samples were sonicated for 15 min in a Branson^®^ sonicator bath (model 2510-MTH, Branson ultrasonic corporation, Danbury, CT, USA), followed by rest for 105 min in refrigeration. Subsequently, the samples were centrifuged at 10,000 rpm for 10 min in a centrifuge (model Z 200 M/H, Hermle-Labortechnik, Wehingen, Germany), decanted, and finally gauged to 2 mL with the same solvent.

#### 3.5.2. Total Soluble Phenol Content

Phenols were quantified with the Folin–Ciocalteu reagent [44] following the method adapted to microplates, onto which 25 µL of extract was placed, and 125 µL of distilled water was added. Subsequently, 20 µL of Folin–Ciocalteu reagent 0.2 N and 30 µL of 20% sodium carbonate (Na_2_CO_3_) were added. The mixture was left to rest for 30 min in the dark, and absorbance was then measured at 760 nm in a plate reader (Epoch, Biotek instruments, Winooski, VT, USA). A standard curve of ferulic acid (R^2^ = 0.995), the most abundant phenolic compound in maize [45], was prepared, and the results were expressed as mg of ferulic acid equivalents (FAE) per 100 g of dry sample (ds).

#### 3.5.3. Total Anthocyanin Content

Anthocyanins were quantified using spectrophotometry, and absorbance was read at 520 nm in a plate reader (Epoch, Biotek instruments, Winooski, VT, USA). A standard curve of pelargonidin chloride (R^2^ = 0.997) (Sigma Aldrich, St. Louis, MO, USA) was prepared since it has been seen that, due to the color of the analyzed hybrids, this compound predominates [18]. A scan was also performed to identify the wavelength of maximum absorbance. The results were expressed in mg of pelargonidin chloride equivalents (PCE) per 100 g of dry sample.

### 3.6. Statistical Analysis

The physical characterization variables of the grains were analyzed in a completely randomized design with two factors of variation: genetic materials and years of cultivation. In the case of flowered grain quality variables, the analysis was performed with a 2 × 11 factorial design, where one factor was the type of flowered grain (with or without pedicel) and the other factor the eleven types of maize evaluated. An analysis of variance followed by comparison of means with the Tukey test (*p* ≤ 0.05) was performed in the case of grain characteristics and phytochemical content. For pozole quality variables, a comparison of measures was made with the Dunnett statistic (*p* ≤ 0.05), using the Cacahuacintle race as a control since it has been the most studied to determine pozole quality [46]. Additionally, Person’s correlation (*p* ≤ 0.05) of the FI with the variables of color and total anthocyanins was made. All measurements were made in duplicate.

## 4. Conclusions

Grain hardness and size of the hybrids Pz1 and Pz2 were modified by effect of crop cycles, while the pink-purple color remained. In the 2019 growing cycle, controlled pollination increased total anthocyanin content relative to that of hybrids with open pollination, but it did not increase the total content of soluble phenols. In 2020 and 2021 growing cycles, the total anthocyanin content was higher than that of hybrids produced with controlled pollination.

In the 2020 growing cycle, where hybrids were studied with their two parents, the parent that participated as female in the cross determined anthocyanin content.

The quality of flowered grain of hybrids was not affected by the presence or absence of the pedicel and was similar to the quality of flowered Cacahuacintle maize grain. However, the presence of pedicel favored a higher phenolic content in flowered grain and flowering broth. In flowered grain, the presence of the aleurone layer was confirmed, which contributes significant amounts of phytochemical compounds to pozole. Hybrids Pz1 and Pz2 have a higher nutraceutical value than Cacahuacintle.

## Figures and Tables

**Table 1:** Grain physical characteristics of pozolero maize NCHs evaluated in the 2019, 2020, and 2021 growing cycles in Celaya, Guanajuato, Mexico.

Hybrid	Year			
	2019	2020	2021	HSD
	Test weight (kg hL^−1^)			
Pz1	74.75 ± 0.7 Aa	71.65 ± 0.35 Ba	68.10 ± 7.35 Aa	17.763
Pz2	73.05 ± 0.07 Bb	73.25 ± 0.35 Aab	73.95 ± 0.07 Aa	0.886
HSD	0.304	1.521	22.375	
	Hundred grain weight (g)			
Pz1	41.92 ± 0.21 Ba	41.04 ± 0.01 Ab	36.38 ± 0.01 Bc	0.512
Pz2	44.51 ± 0.30 Aa	38.59 ± 0.02 Bc	43.24 ± 0.01 Ab	0.736
HSD	1.13	0.068	0.0481	
	Flotation index (%)			
Pz1	58.0 ± 1.4 Bb	88.5 ± 0.7 Aa	93.0 ± 4.2 Aa	10.923
Pz2	68.0 ± 1.4 Aa	79.0 ± 7.1 Aa	82.0 ± 2.8 Aa	18.688
HSD	6.085	21.62	15.513	
	Pedicel (%)			
Pz1	1.32 ± 0.05 Aa	0.85 ± 0.05 Ba	1.37 ± 0.29 Aa	0.719
Pz2	1.23 ± 0.67 Aa	1.23 ± 0.04 Aa	1.19 ± 0.002 Aa	0.199
HSD	0.263	0.198	0.882	
	Pericarp (%)			
Pz1	4.3 ± 0.06 Aa	4.08 ± 0.13 Aa	4.51 ± 0.13 Aa	0.467
Pz2	4.0 ± 0.13 Aa	4.51 ± 0.23 Aa	4.12 ± 0.21 Aa	0.828
HSD	0.444	0.838	0.734	
	Germ (%)			
Pz1	11.35 ± 0.04 Ba	11.49 ± 0.23 Aa	10.39 ± 0.01 Ab	0.572
Pz2	11.77 ± 0.07 Aa	10.99 ± 0.49 Aa	11.13 ± 0.69 Aa	2.072
HSD	0.251	1.684	2.108	
	Floury endosperm (%)			
Pz1	42.5 ± 0.31 Ab	62.26 ± 5.43 Aa	70.67 ± 0.74 Aa	13.245
Pz2	44.13 ± 0.86 Ab	53.11 ± 5.71 Aab	64.78 ± 0.57 Ba	13.992
HSD	2.79	23.967	2.853	
	Vitreous endosperm (%)			
Pz1	40.53 Aa	16.34 Ab	13.07 Bb	13.559
Pz2	38.88 Aa	30.16 Aab	18.78 Ab	13.776
HSD	3.732	23.412	5.668	
	Lightness (%)			
Pz1	48.90 ± 0.04 Aa	41.87 ± 0.28 Bb	48.18 ± 0.82 Aa	2.095
Pz2	46.64 ± 0.35 Bb	46.67 ± 0.16 Ab	50.43 ± 0.74 Aa	1.997
HSD	1.059	0.982	3.352	
	Hue (°)			
Pz1	51.41 ± 0.63 Bc	61.16 ± 0.64 Ab	65.0 ± 1.27 Aa	3.753
Pz2	61.21 ± 0.92 Ab	55.59 ± 1.97 Ab	69.1 ± 2.12 Aa	7.327
HSD	3.372	6.306	7.527	
	Chroma			
Pz1	11.76 ± 0.19 Bb	12.08 ± 10.43 Ab	14.20 ± 0.28 Aa	1.346
Pz2	14.21 ± 0.51 Aa	13.33 ± 0.41 Aa	13.45 ± 0.35 Aa	1.793
HSD	1.662	1.826	1.378	

HSD honest significant difference, capital letters indicate significant statistical difference between hybrids, lower case letters indicate significant statistical differences between crop years according to Tukey test (*p* ≤ 0.05).

**Table 2:** Mean squares and significance in nixtamalization and flowered variables of two pozolero NCHs evaluated in 2019, 2020, and 2021 in Celaya, Gto., Mexico.

		Mean Squares of Technological Variables								
Variation Source	df	YFG	VFG	DLM	FT	MFG	PF	L	Hue	Chroma
Hybrid	10	0.044 *	1607.95 *	0.29	2042.49 *	25.46 *	0.35 *	158.17 *	838.74 *	4.41 *
Flowered	1	0.014	205.11	0.22	9.09	12.767 *	0.039	92.10 *	217.78 *	63.62 *
HXF	10	0.0037	121.36	0.24	409.84	7.61 *	0.099 *	12.36 *	35.21 *	4.56 *
Error	22	0.0043	116.48	0.23	209.95	2.94	0.024	0.26	3.87	0.35
Mean		2.4	354.43	7.33	171.14	61.62	1.56	46.59	54.37	15.49
CV (%)		2.75	3.05	6.48	8.47	2.78	9.99	1.09	3.62	3.82
R		0.84	0.87	0.52	0.84	0.84	0.89	0.99	0.99	0.95

df degrees of freedom, YGF yield of flowered grain, VFG flowered grain volume, DLM dry matter loss, FT flowering time, MFG flowered grain moisture, PF punction force, L lightness, Hue hue angle, Chroma color purity, HXF interaction, CV coefficient of variation, R coefficient of determination. * Significant at 0.05 probability level.

**Table 3:** Flowered quality variables (with and without pedicel) of pozolero NCHs ‘EO’ land-race evaluated in 2019, 2020, and 2021 growing cycles in Celaya, Gto., Mexico.

	FGY (kg of FG Per kg Raw Maize)	VFG (cm^3^)	FT (min)	FGM (%)	DML (%)	FGPF (N)	L (%)	Hue (°)	Chroma
Pz1-19	2.26 ± 0.08 *	332.5 ± 6.45 *	147.5 ± 5.0 *	58.93 ± 1.2	7.23 ± 0.23	1.84 ± 0.07 *	51.92 ± 3.6 *	63.17 ± 3.4 *	16.72 ±2.9
Pz2-19	2.32 ± 0.07	338.8 ± 33.15 *	151.3 ± 10.3 *	57.71 ± 2.9 *	7.71 ± 0.43	1.99 ± 0.2 *	47.53 ± 1.9 *	63.19 ± 3.3 *	14.54 ± 1.9 *
Pz1-19C	2.24 ± 0.11 *	320.0 ± 10.8 *	153.8 ± 7.5 *	57.61 ± 2.5 *	7.32 ± 0.53	1.91 ± 0.15 *	46.21 ± 3.2 *	61.08 ± 3.5 *	15.77 ± 0.5 *
Pz2-19C	2.32 ± 0.07	345.0 ± 10.8 *	150.0 ± 0.0 *	60.29 ± 2.0	7.21 ± 0.49	1.59 ± 0.12	47.52 ± 0.4 *	56.77 ± 3.2 *	15.34 ± 2.4 *
Pz1-20	2.50 ± 0.05	375.0 ± 19.6	196.8 ± 22.6	62.71 ± 0.5	6.88 ± 0.49	1.19 ± 0.11	42.58 ± 1.2 *	40.83 ± 1.9 *	15.11 ± 0.6 *
♀Pz1-20	2.55 ± 0.05	385.0 ± 12.9	194.8 ± 17.04	63.55 ± 0.8	7.70 ± 0.20	1.14 ± 0.21	43.85 ± 1.2 *	46.68 ± 4.1 *	15.74 ± 1.3 *
Pz2-20	2.51 ± 0.07	352.5 ± 8.7 *	161.3 ± 26.3 *	63.73 ± 1.9	7.23 ± 0.48	1.23 ± 0.12	46.76 ±1.1 *	49.34 ± 9.6 *	15.34 ± 0.5 *
♀Pz2-20	2.43 ± 0.02	365.0 ± 10.0	205.0 ± 13.21	62.79 ± 1.2	7.56 ± 0.42	1.61 ± 0.24	40.85 ± 0.4 *	39.08 ± 6.4 *	15.82 ± 0.3 *
Pz1-21	2.38 ± 0.07	351.3 ± 2.5 *	160.0± 14.1 *	63.97 ± 4.0	7.32 ± 0.53	1.52 ± 0.30	42.38 ± 2.3 *	47.68 ± 2.7 *	15.55 ± 0.3 *
Pz2-21	2.41 ± 0.06	356.3 ± 8.5	163.5 ± 16.3 *	64.17 ± 2.9	6.96 ± 0.80	1.76 ± 0.41 *	40.4 ± 5.6 *	41.53 ± 1.9 *	13.21 ± 2.5 *
CACC	2.45 ± 0.05	377.5 ± 8.7	198.8 ± 22.5	62.38 ± 1.0	7.50 ± 0.39	1.41 ± 0.19	62.48 ± 2.4	88.72 ± 2.8	17.21 ± 2.6

FGY flowered grain yield, FG flowered grain, VFG flowered grain volume, FT flowering time, FGM flowered grain moisture, DML dry matter loss, FGPF flowered grain puncture force, L lightness, Hue hue angle, Chroma color purity, ♀ female parent. Means with (*) are statistically different from the control CACC (Dunnett, 0.05).

**Table 4:** Correlation between grain hardness (FI) with color characteristics (L, hue, chroma) and total anthocyanins (TA) in raw maize, flowered grain without and with pedicel of non-conventional hybrids.

	Raw Grain					Flowered Grain Without Pedicel				Flowered Grain With Pedicel			
	FI	L	Hue	Chroma	TA	L	Hue	Chroma	TA	L	Hue	Chroma	TA
FI	1	0.304	0.750 *	−0.279	0.748 *	−0.757 *	−0.819 *	−0.053	0.754 *	−0.506 *	−0.748 *	−0.150	0.730 *
L		1	0.274	0.202	0.015	0.022	−0.125	−0.264	0.225	−0.192	−0.027	−0.150	0.052
Hue			1	−0.317	0.734 *	−0.678 *	−0.539 *	−0.603 *	0.896 *	−0.612 *	−0.692 *	−0.243	0.776 *
Chroma				1	−0.252	−0.066	0.222	0.323	−0.278	−0.064	0.263	−0.253	−0.542 *
TA					1	0.760 *	−0.830 *	−0.214	0.860 *	−0.658 *	−0.840 *	−0.488 *	0.831 *
L_WOP_						1	0.722 *	−0.034	−0.719 *	0.586 *	0.716 *	0.554 *	−0.562 *
Hue_WOP_							1	−0.142	−0.747 *	0.683 *	0.829 *	0.434	−0.804 *
Chroma_WOP_								1	−0.428	0.166	0.082	−0.184	−0.343
AT_WOP_									1	−0.793 *	−0.882 *	−0.437	0.902 *
L_WP_										1	0.833 *	0.477 *	−0.713 *
Hue_WP_											1	0.431	−0.852 *
Chroma_WP_												1	−0.351
TA_WP_													1

* Statistically significant (*p* ≤ 0.05). WOP without pedicel, WP with pedicel.

**Table 5:** Content of total soluble phenols (mg FAE 100 L^−1^ sample) and anthocyanins (mg PCE L^−1^ sample) in nejayote, wash water, and flowering broth from two pozolero NCHs evaluated during 2019, 2020, and 2021 in Celaya, Gto., Mexico.

	Without Pedicel				With Pedicel			
	TSP_n_	TSP_w_	TSP_b_	TA_b_	TSP_n_	TSP_w_	TSP_b_	TA_b_
Pz1-19	316.84 ± 6.2 aA	215.89 ± 7.8 aA	192.73 ± 25.0 abcA	5.14 ±0.2 dA	292.50 ± 5.6 bA	170.67 ± 104 abB	263.93 ± 43.8 aA	4.25 ± 0.1 cB
Pz2-19	314.71 ± 3.6 aA	200.59 ± 15.6 aA	156.55 ± 6.2 bcB	8.00 ± 1.7 cdA	308.02 ± 3.3 abA	145.13 ± 2.4 abB	197.82 ± 2.5 aA	6.17 ± 0.4 abcA
Pz1-19C	324.74 ± 13.4 aA	230.00 ± 2.2 aA	160.45 ± 6.1 bcB	5.04 ± 0.1 dA	314.49 ± 66.7 abA	154.39 ± 1.0 abB	202.73 ± 9.4 aA	4.46 ± 0.01 bcB
Pz2-19C	306.55 ± 0.6 aA	195.59 ± 1.4 aA	152.82 ± 6.5 cB	7.77 ± 0.9 cdA	296.84 ± 3.1 bB	154.88 ± 1.9 abB	197.55 ± 7.3 aA	6.59 ± 0.3 abcA
Pz1-20	316.79 ± 3.1 aB	191.5 ± 16.2 aA	201.38 ± 11.7 abA	12.27 ± 0.2 abA	347.24 ± 6.7 aA	136.50 ± 6.5 abB	203.80 ±7.4 aA	7.27 ± 0.8 abcB
♀Pz1-20	309.74 ± 6.5 aA	201.35 ± 3.5 aA	209.25 ± 7.4 aA	12.76 ± 0.1 abA	316.57 ± 19.7 abA	133.85 ± 6.7 bB	234.96 ± 37.9 aA	8.46 ± 0.9 aB
Pz2-20	316.06 ± 1.3 aA	197.53 ± 4.2 aA	196.79 ± 10.6 A	11.00 ± 0.6 bcA	309.74 ± 3.5 abA	175.42 ± 9.9 abA	239.78 ± 41.3 aA	7.01 ± 2.1 abcA
♀Pz2-20	324.78 ± 24.7 aA	208.85 ± 23.7 aA	188.04 ± 5.9 abcA	14.90 ± 0.2 aA	327.75 ± 2.8 abA	159.88 ±26.2 abA	212.28 ± 28.2 aA	8.14 ± 0.7 abB
Pz1-21	297.24 ± 7.1 aA	193.78 ± 17.8 aA	198.03 ± 7.6 abcB	10.30 ± 2.4 bcA	321.35 ± 22.9 abA	175.32 ± 5.9 abA	239.39 ± 10.1 aA	5.82 ± 0.9 abcB
Pz2-21	298.36 ± 10.2 aA	179.81 ± 22.8 aA	190.15 ± 17.7 abcA	9.94 ± 0.7 bcA	306.45 ± 3.1 bA	145.18 ± 5.8 abA	226.70 ± 41.1 aA	6.34 ± 1.7 abcB
CACC	321.25 ± 1.5 aA	220.76 ± 3.33 aA	167.41 ± 9.1 abcB	0.0 ± 0.0 eA	297.43 ± 7.8 bA	178.90 ± 17.3 aA	205.54 ± 20.9 aA	0.0 ± 0.0 dA
HSD	38.72	52.44	46.8	3.88	40.34	44.05	108.8	3.82

TSP total soluble phenols, TA total anthocyanins, n nejayote, w wash water, b flowering broth, HDS honest significant difference, ♀ female parent. Means with different lowercase letters indicate significant statistical differences between genotypes, and means with different capital letters indicate statistical differences between treatments (without and with pedicel), according to Tukey statistic (*p* ≤ 0.05).

## Data Availability

The data presented in this study are available within the article.
